# On the probability of dinosaur fleas

**DOI:** 10.1186/s12862-015-0568-x

**Published:** 2016-01-11

**Authors:** Katharina Dittmar, Qiyun Zhu, Michael W. Hastriter, Michael F. Whiting

**Affiliations:** Department of Biological Sciences, University at Buffalo, Cooke 109, Buffalo, NY 14260 USA; Monte L. Bean Museum, Brigham Young University, 336 MLB, Provo, UT 84602 USA; Department of Biology and M. L. Bean Museum, Brigham Young University, 4142 LSB, Provo, UT 84602 USA; Graduate Program of Evolution, Ecology, and Behavior, University at Buffalo, State University of New York, 411 Cooke Hall, Buffalo, NY 14260 USA

**Keywords:** Fleas, Dinosaurs, Ectoparasites, Siphonaptera

## Abstract

Recently, a set of publications described flea fossils from Jurassic and Early Cretaceous geological strata in northeastern China, which were suggested to have parasitized feathered dinosaurs, pterosaurs, and early birds or mammals. In support of these fossils being fleas, a recent publication in BMC Evolutionary Biology described the extended abdomen of a female fossil specimen as due to blood feeding.

We here comment on these findings, and conclude that the current interpretation of the evolutionary trajectory and ecology of these putative dinosaur fleas is based on appeal to probability, rather than evidence. Hence, their taxonomic positioning as fleas, or stem fleas, as well as their ecological classification as ectoparasites and blood feeders is not supported by currently available data.

Siphonaptera (fleas) is a clade of blood-feeding holometabolous insects. Monophyly has been demonstrated by taxonomic, as well as molecular studies [1, 2]. Taxonomically, clade membership is conferred if, and only if all of the following character complexes are present: a) a latero-lateral compression of the body, b) winglessness with a modified wing joint (pleural rod, and pleural arch), with resilin, and enlarged metacoxae and metafemorae, and c) the presence of a saddle-shaped sensilial plate (pygidium), dorsally placed on sternum X. Within the universe of extant insects this character combination is unique to fleas [2, 3].

Ecologically, Siphonaptera are referred to as ectoparasites [4, 5]. This form of exploitative symbiosis includes obtaining nutrients (blood) directly from mammal or bird hosts, the presence of adult stages on the surface of the host’s body, as well as the expression of adaptive structural modifications facilitating the maintenance of a spatial relationship with a host (e.g., ctenidia on the body, claws).

Recently, the morphology of a set of Middle Jurassic and Early Cretaceous wingless invertebrate compression fossils with siphonate mouthparts from North Eastern China has been interpreted as indicative of fleas or stem-fleas (=Mesozoic fossils, hereafter) [6–9]. Following this initial argumentation, the claim of ectoparasitic host association with feathered dinosaurs, pterosaurs and early birds or mammals has been forwarded, simply based on the discovery of their fossil representatives in contemporaneous geological formations. Finally, the extended abdomen of one Mesozoic fossil was interpreted as sign of a blood meal, which, according to the authors, establishes trophic association with a terrestrial vertebrate, and cements the argument of these fossils being ectoparasites [10].

While an interesting scenario to ponder, upon closer consideration of the presented evidence, several issues surface that challenge this argumentation.

**First,** none of the Mesozoic fossils show the diagnostic character combination required to place them within extant Siphonaptera (i.e. fleas). Among all Mesozoic fossils only Pseudopulicidae sensu Gao et al. (2012) seem to present a sensilial plate with well defined sensory pits. However, the anatomical position of this putative pygidium is unclear, and seemingly alternates between a ventral [6] or dorsal abdominal position [10] depending on publication. All Mesozoic fossils exhibit a dorso-ventrally flattened, or cylindrical body aspect, and although all specimens are wingless, the third pair of legs shows no concurrently modified metacoxae and metafemorae, and no structures suggesting the presence of a modified wing joint.

All other morphological characters cited to overlap with those of extant fleas, such as posteriorly directed setae and bristles, reduced eyes (in some specimens), comb-like structures on legs, claws with scythe-like appearance, and winglessness are known to be convergent characters throughout extant and extinct Insecta. Therefore, in absence of any further systematic phylogenetic analysis, these characters should not be used to support (or refute) shared ancestry with Siphonaptera. Additionally, recent molecular analyses support Cretaceous beginnings for all extant siphonapteran lineages, which would then overlap in age with the morphologically very distinct Cretaceous fossils [11, 12].

In essence, at this point there is no taxonomic premise to suggest these fossil lineages are fleas, are ancestors of fleas, or ever transitioned into fleas.

**Second,** following from the presence of robust siphonate, piercing mouthparts with serrating stylets it is reasonable to assume that the Mesozoic fossils penetrated a surface to extract a liquid diet. However, the claim of blood-feeding is based on an unfounded a priori taxonomic assumption (=they are fleas), and a selective interpretation of observing abdominal expansion in one female Mesozoic fossil [10]. In fact, the dietary proclivities of these Mesozoic fossils are currently unknown. Based on the range of known diets in extant insects with piercing-sucking mouthparts the fossils could have fed on plants (phloem), vertebrates (blood), or even larger invertebrates (hemolymph).

Moreover, none of the eight newly minted Mesozoic putative fleas have been described in the context of a vertebrate host or at the very least, their general death assemblage. This, in connection with their uncertain taxonomic position, makes any claim of a trophic association with, and habitat restriction to feathered dinosaurs, pterosaurs, and early birds or mammals mute, even if these vertebrates have a representation in the same general geological formation as the Mesozoic insect fossils. The fundamental problems with such argumentations have already been discussed extensively in the paleontological literature [13].

Finally, many ectoparasites show convergent structural modifications in distantly related insect clades; e.g. some Siphonaptera, hippoboscoid flies, and Polycteniidae have ctenidia and/or reduced eyes [1, 14, 15]. However, the observation of these modifications out of any verifiable ecological context does not provide proof of an ectoparasitic lifestyle. This is because these structural solutions are not unique to ectoparasites (see taxonomic premise), and may arise in diverse ecological settings. For instance, eye reduction may arise from living in aphotic environments, or due to a lacking need for visual mate recognition [16, 17]. As another example: long, scythe-like claws are by no means unique to mammalian ectoparasites, but are routinely observed in any insect navigating complex three dimensional terrains [18].

In summary, there is no ecological premise to categorize any of these Mesozoic fossils as blood-feeders, or ectoparasites.

## Conclusions

Given the uncertainty of taxonomic and ecological data, the interpretation of the Mesozoic fossils as blood-feeding, ectoparasitic dinosaur fleas is a non-sequitur, based on the logical fallacy: It might be possible, and therefore it is true. This unsupported interpretation has potentially far reaching implications by generating a biased view of not only flea evolution, early insect evolution and age, but also dinosaur or pterosaur ecology (by claiming a species interaction with blood-feeding fleas), as well as pathogen evolution (fleas are vectors).

Our alternative, and decidedly more conservative conclusions are as follows: Eight new species of Mesozoic insects were recently documented. While none of these fossils can be intuitively connected to any extant clade of Insecta, the consistent presence and specific morphology of their siphonate mouthparts make a relationship to alate siphonate Mecoptera, such as Aneuretopsychidae, or to apterous Siphonaptera possible [19], but further systematic studies are needed to elucidate their phylogenetic placement within Insecta. These insects likely fed by suctorial uptake of a liquid diet.

## Response

By Taiping Gao^1^, Alexandr P. Rasnitsyn^2, 3,*^, Chungkun Shih^1^, Xing Xu^4^, Shuo Wang^1^ and Dong Ren^1,*^

1. College of Life Sciences, Capital Normal University, 105 Xisanhuanbeilu, Beijing 100048, China

2. Palaeontological Institute, Russian Academy of Sciences, Moscow 117997

3. Natural History Museum, Cromwell Road, London SW7 5BD, UK

4.  Institute of Vertebrate Paleontology and Paleoanthropology, Key Laboratory of Evolutionary Systematics of Vertebrates, Chinese Academy of Sciences, 142 Xiwai Street, Beijing 100044, China.

* Corresponding author, Email: alex.rasnitsyn@gmail.com, rendong@mail.cnu.edu.cn

The authors thank Dittmar et al. for their interesting and stimulating Correspondence questioning the identifications of the Mesozoic ‘fleas’ [6–10] and providing alternative interpretations. However, their rebuttal is misaddressed at least in part, because the hypothesis and recent reports in question have never implied attribution of the respective fossils to the flea crown group, thus making irrelevant the fact that these fossils lack apomorphies of the flea crown group, as listed under ‘**First**’ by Dittmar et al. They expect that stem fleas should have a whole suite, or most, of features seen in crown fleas (eg. dorsal-ventrally flattened body in stem fleas vs. laterally flattened body in extant fleas, etc), which is fundamentally against the character distribution pattern (step-wise acquisition) seen in all known life lineages.

Dittmar et al. used their result of Cretaceous diversification of crown fleas based on molecular clock against the identification of the stem fleas reported from the Mid Mesozoic. This is also inadvisable because the stem fleas are significantly different from the crown fleas, and they are highly likely to have co-existed with the crown fleas. In addition, we take exception to their misleading and dramatized terminology of “dinosaur fleas” in their title and text that we have never used in our papers. We also want to clarify a misguided term of “siphonate mouthparts” used by them. There are clear and significant distinctions, morphologically and functionally, between “siphonate sucking mouthparts” and “piercing-and-sucking stylet mouthparts” [6].

To proceed further, let us reiterate the evidence in favor of the flea affinity of the fossil insects under question. These Mesozoic ‘fleas’possess the following features: the piercing-and-sucking beaks with thick serrate stylets imply a liquid feeding through rather thick and tough body coverings; the soft and distensible abdomen of the female might suggest their adaptation to intake large amount of food at once; relatively large and wingless body with long but thin legs and long and sickle-shaped claws imply that they adapt to live on a large surface; and body and legs covered with stiff spines and setae in well-organized fashion, all directed backward, suggestive of adaptation to fix and move on a surface covered with hairs or feathers.

Each of the above features separately can be probably found in some insects. However, for insects having all these characters in combination, in our opinion, they should be adapted to blood sucking of vertebrate host with outgrowths like hairs or feathers. These features in combination are not consistent with all the known herbivorous insects. Ponomarenko [20] considered this group as pterosaur parasitism first. We believe any sufficiently big mammals, birds, feathered dinosaurs and pterosaurs with partially naked, elastic skin with hairs, feathers and the like, are possible hosts of parasitism by saurophthirids, pseudopulicids and tarwiniids [6, 7, 10].

Concerning the taxonomic position of the above three taxa, we again start with their relevant features:

- A combination of sucking stylet beak, 5-segmented tarsi and complicated, symmetrical male genitalia easily homologizible to those in Hymenoptera and Mecoptera ([21] and [10]) implies the holometabolan ancestry of these insects. Among the Mesozoic holometabolan insects, their homonomous pterothoracic segments exclude placement into Hymenoptera, Coleoptera and Diptera. Neuropteroids do not have any specific similarity, any adult parasitism or any adult adaptation to prefer liquid diet (siphonate beak included [22]), resulting in exclusion of their placement into Neuropteroidea;
- Within Mecopteroidea, close affinity to Diptera is most unlikely because of the homonomous pterothorax. Amphiesmenoptera have mandibles reduced or, exceptionally, chewing (never piercing), and sucking siphonate beak (never piercing) when present, and otherwise show no specific similarity to above three taxa;
- Within Mecoptera, the clade Aneuretopsychina is generally considered the most likely relative of the Mesozoic ‘fleas’. This idea is based solely on the very presence of a beak, in spite that Aneuretopsychina have well-elaborated siphonate sucking mouthparts or, only in the most advanced Pseudopolycentropodidae, piercing-and-sucking beak [23], which is thin, delicate, lacking robust and serrate stylets. We are not to rebut their hypothesis but to direct the attention to the fact that this implies position of the Mesozoic ‘fleas’ at most as a sister group of Aneuretopsychina (Fig. three in [10]) and not as a part of it. Even if the sister group relations is correct, this does not rule out their relation to fleas;
- Specifically similar features of the Mesozoic ‘fleas’ to the crown fleas are not many nor unquestionable, but they do exist. One of these is the most likely blood sucking on non-scaled skin of amniote vertebrates (see above). The other possible synapomorphy is the comparable beak construction with serrate stylets and palps of comparable length and forming rather loose stylet sheaths. The third possible synapomorphy is the compact antenna with short, bell-like flagellar segments. The fourth is a pygidial plate known in Pseudopulicidae only and well identifiable there as dorsal (Figs. two(e) and three(b) in [9]; Fig. one(a) in [6]; Figs. one(a) and (j) in [10]).

Even though the above evidence and rationale for supporting the Mesozoic ‘fleas’ to form a stem group of the modern fleas are not conclusive, we deemed it is worthwhile to put forward as a proposed phylogeny [10] with the hope that future new fossil specimens and new studies will shed more lights to enhance our knowledge. The proposed phylogeny is not necessarily contradictory to evidence of affinity between fleas and Boreidae, which is in turn not unquestionable (the proposals are strictly different in [1], [24] and [11]) and which is based on a different set of characters and does not consider fossils. In summary, we will be happy to see an alternative hypothesis, equally plausible and evidence-backed, of flea ancestry, whenever it is available.
